# Bioactive folate attenuates valproic acid-induced NAFLD in female rats

**DOI:** 10.1186/s12876-026-05047-2

**Published:** 2026-06-30

**Authors:** Hajra Ashraf, Tooba Asif, Fatima Baqir, Naila Naz

**Affiliations:** Department of Biomedicine, Atta-ur-Rahman School of Applied Biosciences, National University of Sciences & Technology, Islamabad, Pakistan

**Keywords:** Nonalcoholic fatty liver disease, Sodium valproate, Hepatic steatosis, Drug-induced liver injury, Bioactive folates, Nrf2-KEAP1 pathway, Oxidative stress, Inflammation, Molecular docking, Hepatoprotection

## Abstract

**Background:**

Nonalcoholic fatty liver disease (NAFLD) is a key contributor to chronic liver diseases. It is defined as hepatic fat accumulation in ≥ 5% of hepatocytes in individuals with minimal or no alcohol consumption and no other identifiable causes of liver steatosis. Drug-induced hepatic toxicity is increasingly recognized as a key contributor to the development and progression of NAFLD. Among them, valproic acid (VPA), an antiepileptic drug, is well documented for inducing hepatic steatosis, in addition to interfering with folate metabolism and accelerating the progression of NAFLD. The current study distinctively evaluates the protective role of bioactive folate derivatives (folinic acid and 5-methyltetrahydrofolate) in a VPA-induced NAFLD rat model.

**Methods:**

Adult female Sprague Dawley rats were divided into three groups: control, diseased and treated. Liver function test, histopathological examination, gene expression analysis, and molecular docking were performed.

**Results:**

Liver function tests indicated that the increase in alanine aminotransferase, alkaline phosphatase, and total bilirubin levels was highly significant in the diseased group, indicative of hepatic injury. Histopathological examination demonstrated severe steatosis in VPA-treated animals, which was significantly improved in the folate-treated group. The NAFLD Activity Score, a mild NAFLD was observed in the diseased group with a total score of 2. Gene expression studies showed high upregulation of HO-1 and pro-inflammatory cytokines TNF-α and IL-6 in the diseased group, whereas downregulation was observed in folate-treated rats. Furthermore, molecular docking studies indicated that folinic acid and 5mTHF showed high affinities for binding to KEAP1 protein, suggestive of activation of the Nrf2-KEAP1 antioxidant pathway.

**Conclusions:**

Collectively, our data provide significant evidence for the protective potential of bioactive folate supplementation in the amelioration of the VPA-induced liver injury through modulation of oxidative stress, inflammation, and lipid accumulation.

## Background

Nonalcoholic fatty liver disease (NAFLD) refers to a chronic state of the liver; it is becoming more prevalent due to excessive deposition of fat in the hepatocytes without any remarkable consumption of alcohol. NAFLD is clinically classified into two major classes: (i) nonalcoholic fatty liver, which usually evidences simple steatosis; (ii) nonalcoholic steatohepatitis, which includes steatosis and hepatic inflammation. Left unattended, it may progress toward causing severe liver ailments, such as fibrosis, cirrhosis, or even hepatocellular carcinoma [1, 2]. The development of NAFLD depends on many risk factors, including obesity and insulin resistance, metabolic syndrome, therapeutic drugs, and micronutrient deficiency, including folates [1, 2, 3]. Worldwide, roughly 30.2% of people are afflicted with this disease, whereas alone, the regional prevalence in Asia is at 30.9% [1], where in Pakistan, NAFLD stands at 29.8% [4]. 

Valproic acid, or VPA, is an antiepileptic and a broad-spectrum mood stabilizing medication. In regular clinical practice, it is used as a reserve treatment, not always the first choice. Recently, it has been increasingly recognized to induce NAFLD [5]. Among those patients who have received long-term VPA treatment, it is estimated that 61% have developed hepatic steatosis. Among non-obese epileptic patients, the prevalence of NAFLD was shown to reach a staggering 60.9% with VPA treatment in a cross-sectional survey; impressively higher than that of CBZ or LTG patients or those on any other antiepileptic. Though incompletely understood, mounting evidence shows that VPA disrupts lipid metabolism, causes mitochondrial dysfunction, impairs activity of the bile salt export pump, and greatly interferes with folate metabolic pathways [6–9]. However, VPA-induced upregulation of CD36 and DGAT2 provides clearer insight into the mechanisms underlying VPA-induced hepatic steatosis [6, 10].

VPA impairs the bioavailability of folate by reducing its gastrointestinal absorption, increasing renal excretion, and affecting many enzymes involved in one-carbon metabolism, including methylenetetrahydrofolate reductase and methionine synthase [11]. Such interruptions lead to disturbed DNA methylation, altered gene expression, and accumulation of hepatic lipids, which can worsen the pathology in NAFLD [12]. Further support for the idea that a dietary or drug-induced folate deficiency could worsen fatty liver disease is provided by interaction between VPA and folate metabolism genes [13]. Folic acid or vitamin B9 is an important water-soluble micronutrient deemed important in one-carbon metabolism, the biochemical network linking nucleotide synthesis, DNA methylation and methylation of proteins, interconversion of amino acids, and antioxidant-defense mechanisms. Folate becomes converted in part via vitamin B6-and B12-dependent enzyme pathways into THF and 5-mTHF, which are its bioactive forms [14, 15]. A previous study reported that these bioactive folate derivatives were thought to be the major contributors in regulating hepatic classes of lipid metabolism, redox balance, and epigenetic integrity [2].

There has been some fundamental evidence supporting the close relationship between folate deficiency and the aggravation of NAFLD. High dietary folate and B vitamin intake may decrease the risk of NAFLD, probably because of their role in one‑carbon metabolism and adequate regulation of lipid homeostasis in the liver [16]. Folates play an important role in the methionine cycle and maintain the SAM and homocysteine levels, which are altered during hepatic steatosis [17]. Recently, an increasing amount of evidence points out that deficiency or impairment of the folate-dependent one-carbon metabolic pathway interferes with lipid metabolism, thereby decreasing the synthesis of phospholipids (especially phosphatidylcholine) and inhibiting VLDL-mediated lipid export, contributing to NAFLD and hepatic steatosis [18]. Besides that, the status of other associated nutrients could possibly be low, namely methionine and choline, both of which are essential for the folate cycle, contributing to fat deposition and oxidative stress in the liver.

In light of this background and knowledge, the current study was designed to evaluate whether bioactive folate supplementation, in particular, a combination of folinic acid and 5-methyltetrahydrofolate, would offer protection to the liver under VPA-induced NAFLD in an experimental model using adult female rats, mainly through histopathological, molecular, and in silico routes.

## Methods

### Animal selection criteria

Rats were selected primarily because of their well-established hepatotoxicity and their frequent use in NAFLD-related work. In practice, rats provide a sufficient blood volume for the biochemical assays, and on top of that, they usually show fairly low within-group variability between individuals, so the readouts stay kind of stable. VPA was administered intraperitoneally, mainly to ensure consistent systemic exposure and to allow for repeatable hepatic injury induction across animals. To maintain biological consistency and to limit inter-sex hormonal differences that can otherwise create variable outcomes, only female rats were used. Rats of approximately 8–10 weeks of age, weighing approximately 250–350 g, were collected from the National Institute of Health, Pakistan. Healthy rats. Healthy rats, without any apparent disease or injury, were included.

### Ethical approval

The protocol was reviewed and approved by the Institutional Animal Ethics Committee, approval number: 2025-IRB-A74/74, with an approval date of 21-2-2025. The rats were kept under standard lab conditions (temperature 20–25 0 C, humidity 30–50%, and a 12 h light/dark cycle) with free access to food and water. Throughout the study, every effort was made to reduce pain and distress as much as possible.

Animals were observed daily during the whole experimental period for any signs of pain, distress, or a drop in health status, in line with ARRIVE guidelines. The humane endpoints were set ahead of time and included clear body weight loss (more than 15% of the original body weight), persistent lethargy, diminished mobility, inability to reach food or water, marked breathing distress, abnormal behavior, seizures, self-injury, or, basically, any declining situation that suggested serious suffering. If an animal reached any of these conditions, it was meant to be removed from the study and euthanized humanely, so the overall pain and stress stayed as low as feasible. At the same time, all welfare assessments and any necessary interventions were handled according to institutional animal care rules, plus the AVMA Guidelines for the Euthanasia of Animals (2020 edition). At study start, baseline features such as sex, age, body weight, and health status were comparable across all groups. During the entire period, animals were checked daily for signs of distress or treatment-related problems, and there were no mortality events, no adverse events, and no exclusions connected to humane endpoints.

### Research design

The researchers grouped the animals into groups blindly. All experimental conduct, outcome assessment, and histopathological evaluation were performed in a blinded manner to reduce observer bias during data analysis. As the current study is pilot and exploratory, a group size of 12 (four rats per group) was selected, consistent with exploratory/pilot NAFLD animal studies and in line with the ARRIVE principles of reduction, where small cohorts are acceptable for preliminary mechanistic investigations prior to adequately powered confirmatory studies [19]. All animals completed the study and were included in the final analyses.

### Animal grouping

The study included 3 groups. Group 1: healthy control group; Group 2: diseased group (VPA-induced NAFLD); and Group 3: diseased treated group (folate + VPA treatment).

### Animal model induction

All compounds used in the study were prepared as aqueous solutions and all injections were made intraperitoneal (IP). The healthy control group received saline (Sigma Aldrich, USA) injection. The diseased group received VPA (Epival, Abbott, Pakistan) in two doses of 600 mg/kg given 7 h apart making it a total dose of 1200 mg/kg/day) as described earlier [6]. Animals in the treatment group were given a combination of folinic acid (Pfizer, USA) and 5-methyltetrahydrofolate (MedChemExpress, USA) in a dose of 2.25 mg/kg ^6,20^. To prepare this dose both folates were used in 1:1 ratio i.e. 1.125 mg/kg of folinic acid and 1.125 mg/kg of 5mTHF. Combination therapy was started 1 week before the administration of VPA and continued for 10 days after VPA exposure.

### Sample collection

At the end of the study, animals were humanely euthanized using IP administration of ketamine (CUKET, Cunningham Pharmacuticals Pvt. Ltd, Pakistan) at a dose of 90 mg/kg body weight, in accordance with the AVMA Guidelines for the Euthanasia of Animals (2020 edition). The corneal reflexes and the absence of pedal withdrawal confirmed adequate depth of anesthesia. Following confirmation of complete unconsciousness, euthanasia was carried out by cervical dislocation to ensure death. Tissues and blood samples were collected. The blood was drawn from the aorta into EDTA-coated vacutainers (BD: Becton, Dickinson and Company, USA). The liver tissues were dissected and separated for storage: half were snap-frozen in liquid nitrogen and kept at -80 °C for molecular analyses, and the other half were fixed in 4% paraformaldehyde (PFA, Sigma Aldrich, USA) for histological studies.

### Primary and secondary outcome

Primary outcome measures were the serum profile and the histopathological evaluation of liver tissue, mainly through steatosis assessment, lobular inflammation, hepatocellular ballooning, and the overall NAFLD Activity Score NAS. For the secondary outcome measures, qRT-PCR was performed for HO-1, IL-6, and TNF-α gene expression, and a molecular docking study of bioactive folates with KEAP1.

### Liver function tests 

Whole blood was centrifuged at 3000 rpm for 10–15 min. to separate the serum. Serum was assayed for various liver function parameters, including ALT, ALP, and total bilirubin, using commercially available kits: ALT (GPT), IFCC method; ALP, DGKC method; and total bilirubin, Jendrassik-Grof method. Absorbance was read on a MICROLAB 300-automated clinical chemistry analyzer (Vital Scientific, Netherlands).

### Histological analysis of the liver

The fixed liver tissues were dehydrated in graded ethanol (Sigma Aldrich, USA), cleared in xylene (Sigma Aldrich, USA), paraffin-impregnated (Sigma Aldrich, USA), and sectioned on a rotary microtome on glass slides (Leica Biosystems, USA). Tissue sections were stained with hematoxylin and eosin (H&E, Diachem, China) for light microscopic examination at 10× and 40× magnifications. Image capture was completed with OPTIKA Vision Lite 2.1 software. Steatosis, lobular inflammation, and hepatocyte ballooning were monitored according to the NAS.

### RNA extraction and cDNA synthesis

TRIzol reagent (Thermofischer Scientific, USA) was used to extract total RNA from the frozen liver tissues as described [21]. RNA purity and concentration were determined by the NanoDrop ND-1000 spectrophotometer, version 3.8.1. cDNA was synthesized by reverse transcription using a commercially available kit (Bio-Rad, USA) according to the manufacturer’s instructions.

### RT-PCR

Quantitative RT-PCR was performed to study the expression of the pro-inflammatory and oxidative stress markers IL-6, TNF-α, and HO-1 as per the protocol published before [20]. For normalization, GAPDH was used as a housekeeping gene. Primers used in this study are listed in Table 1.

**Table 1:** Primer sequences for real-time polymerase chain reaction (RT-PCR)

Gene	Forward Primer (5’–3’)	Reverse Primer (5’–3’)
HO-1	CACAAAGACCAGAGTCCCTCACAG	AAATTCCCACTGCCACGGT
IL-6	ACCAAGACCATCCAACTCATC	TTCTGACCACAGTGAGGAATG
TNF-α	ACTGAACTTCGGGGTGATCG	TGCTTGGTGGTTTGCTACGA
GAPDH	CATCACTGCCACCCAGAAGACTG	ATGCCAGTGAGCTTCCCGTTCAG

Primer sequences were made for quantitative real-time PCR readout, and they are shown in the 5′–3′ direction

*HO-1 *Heme oxygenase-1, *IL-6* Interleukin-6, *TNF-α *Tumor necrosis factor-alpha, *GAPDH *Glyceraldehyde-3-phosphate dehydrogenase

### Molecular docking 

Molecular docking studies were carried out to assess the interaction of bioactive folate compounds with the Keap1 protein, a regulator of oxidative stress pathways. The 3D structure of KEAP1 (pdb_00006hws) was retrieved from the RCSB Protein Data Bank, while the molecular structures of 5-mTHF (CID: 135398561) and folinic acid (CID: 135403648) were sourced from PubChem. Docking simulations were performed using PyRx virtual screening software. Binding affinities and interaction poses were analyzed to predict the potential of folate ligands to modulate Keap1 activity.

### Statistical analysis

Data are expressed as mean ± standard deviation (SD). The comparisons between the experimental groups were performed by one-way analysis of variance (ANOVA), and subsequently, the appropriate post hoc multiple comparison tests were applied. The level of significance was set to *p* < 0.05. The histopathological scores and molecular data were processed separately depending on the specific experimental objectives. There were also no additional statistical adjustments or data transformations, as the data apparently met the assumptions for parametric analysis. The number of animals used for each analysis is explicitly reported. All available data were used, and nothing was excluded.

## Results

### Bioactive folates reverse VPA-induced hepatic injury

**Fig. 1:**
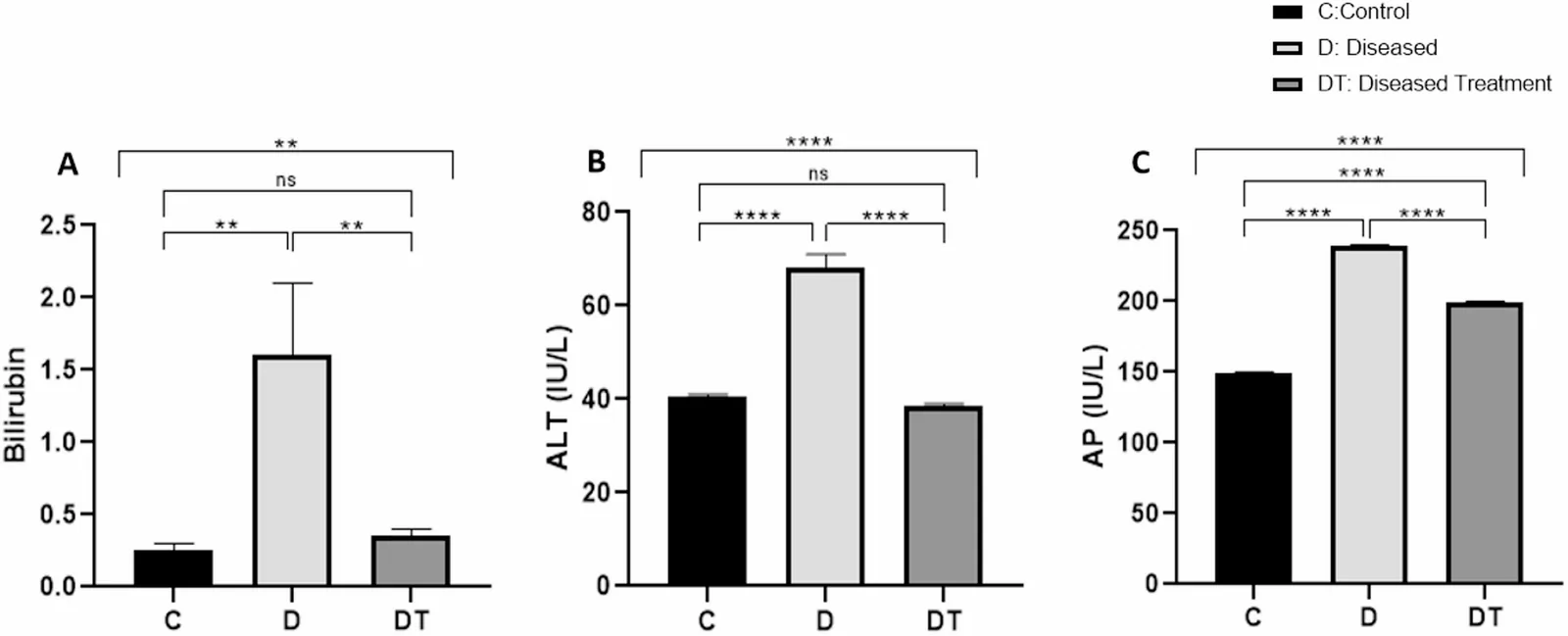
Bar graph depicting liver function test. **A**: Total bilirubin in mg/dl, **B**: Alanine transferase (ALT) in IU/L, **C**: Alkaline phosphatase (AP) measured in IU/L in control (C), diseased (D), and a diseased treated (DT). *n* = 3. ns = non-significant, **p < 0.01, (****p < 0.0001)

LFT indicated significantly raised levels of ALP, ALT, and total bilirubin in the diseased group, thus indicating hepatic injury and impairment of liver function. These results are graphically illustrated in Fig. 1. Graph A: Total bilirubin is seen to be significantly *p* < 0.01elevated (1.60 ± 0.17) within the diseased group as compared to the healthy controls (0.24 ± 0.10), which indicates that the hepatic ability of processing and excreting bilirubin is compromised; however, there was a significant reduction of bilirubin levels (0.35 ± 0.17) within the diseased treatment group, which suggested a return of liver function. Graph B: The same trend is extended in Graph B to ALT level, which was significantly (*p* < 0.0001) elevated (68 ± 1) in the diseased group and reflects hepatocellular damage. In addition, ALT in the treated group was reduced to 38.5 ± 0.17 and nearly reached the control group. Figure C: In Graph C, ALP levels are shown to be significantly (*p* < 0.0001) increased in the diseased group (239 ± 0.33), indicating cholestatic injury and hepatotoxicity. Bioactive folate supplementation reduced ALP levels in the treatment group (199.5 ± 0.17), demonstrating its hepatoprotective role.

### Bioactive folates reverse VPA-induced hepatic steatosis

**Fig. 2:**
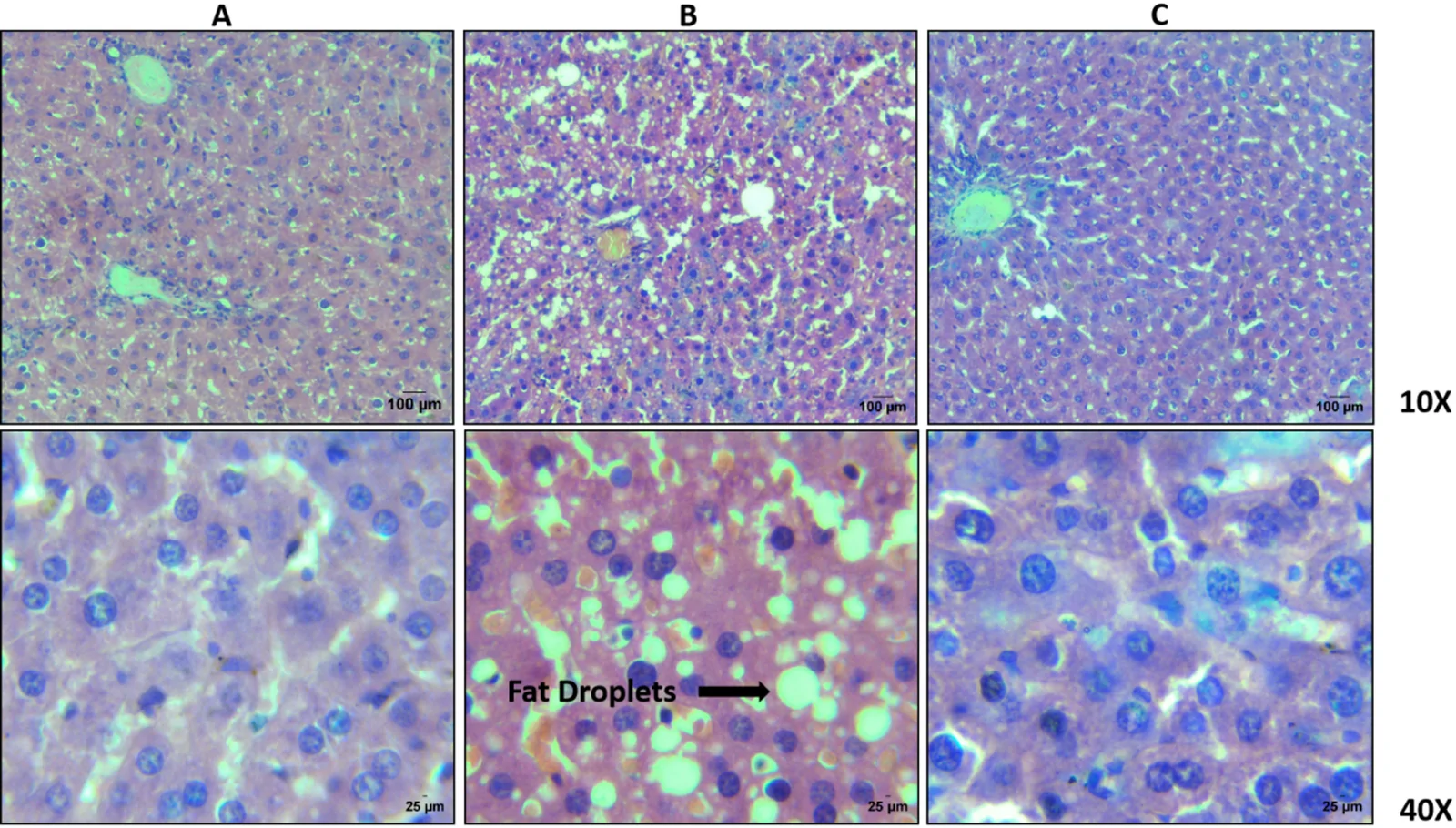
Hematoxylin and eosin staining of hepatic section. **A**: Control: Normal morphology of liver. **B**: valproic acid (VPA) treated diseased group and **C**: Disease Treatment group (VPA + Folinic acid + 5mTHF). This result is representative for *n* = 3. Magnification of the top panel is 10x at a scale bar of 100 μm, and the bottom panel is a 40x image of the same area with a 25 μm scale. The black arrow indicates the presence of fat droplets

Hepatic sections from the control group show normal histological architecture, manifested in intact hepatocytes, clear sinusoidal spaces, and well-defined central veins in liver tissue (Fig. 2A). The diseased group showed quite noticeable histopathological changes of steatosis as evident from the presence of fat vacuoles within the hepatocytes, both at 10× and 40× magnifications, confirming the VPA-induced lipid accumulation (Fig. 2B). Liver tissue in Fig. 2C from the diseased treatment group demonstrated a significant amelioration. Generally, its hepatic morphology is similar to that of the control group, which manifested a lower degree of steatosis but suggested the restoration of normal morphology, thus showing possible protective effects of bioactive folate supplementation.

### Bioactive folates attenuate NAFLD severity 

The NAS is a histological score used to validate grading NAFLD and its severity, taking into account three main histological features of the disease: steatosis, lobular inflammation, and hepatocellular ballooning. The control group was assigned a total score of 0 and was used as a reference. In the diseased group, NAS scoring revealed some degree of steatosis and scored 1, representing moderate fat accumulation within hepatocytes. Lobular inflammation scored 1 (only a few inflammatory cells in the hepatic lobules), and no features of hepatocellular ballooning were seen, scoring 0. Thus, total NAS is 2, indicating that rats indeed suffered from mild NAFLD. Histopathological examination of the folate-treated group demonstrated marked improvement in morphology with absence of steatosis and lobular inflammation, and no hepatocellular ballooning (NAS score 0), therefore indicating complete resolution of fatty liver changes in the treated rats (Table 2).

**Table 2:** Effect of valproic acid (VPA) and bioactive folate treatment on non-alcoholic fatty liver disease (NAFLD) activity score (NAS) in female rats

Group	Steatosis Score (Mean ± SEM)	Lobular Inflammation Score (Mean ± SEM)	Hepatocellular Ballooning Score (Mean ± SEM)	Total NAS Score (Mean ± SEM)	NAFLD Severity
Control	0.00 ± 0.00	0.00 ± 0.00	0.00 ± 0.00	0.00 ± 0.00	Normal
Diseased (VPA)	1.00 ± 0.21	1.00 ± 0.18	0.00 ± 0.00	2.00 ± 0.26	Mild NAFLD
Treated (VPA + Folate)	0.17 ± 0.11	0.17 ± 0.11	0.00 ± 0.00	0.33 ± 0.21	Normal / Resolved

The NAFLD activity score was determined based on histopathological assessment of steatosis, lobular inflammation, and hepatocellular ballooning. A higher NAS indicates greater severity of hepatic injury. Bioactive folate treatment improved liver histology and reduced NAS in VPA-induced NAFLD female rats

*NAS* Non-Alcoholic Fatty Liver Disease Activity Score, *VPA *valproic acid

### Bioactive folates attenuate NAFLD-associated hepatic oxidative stress and inflammation

**Fig. 3:**
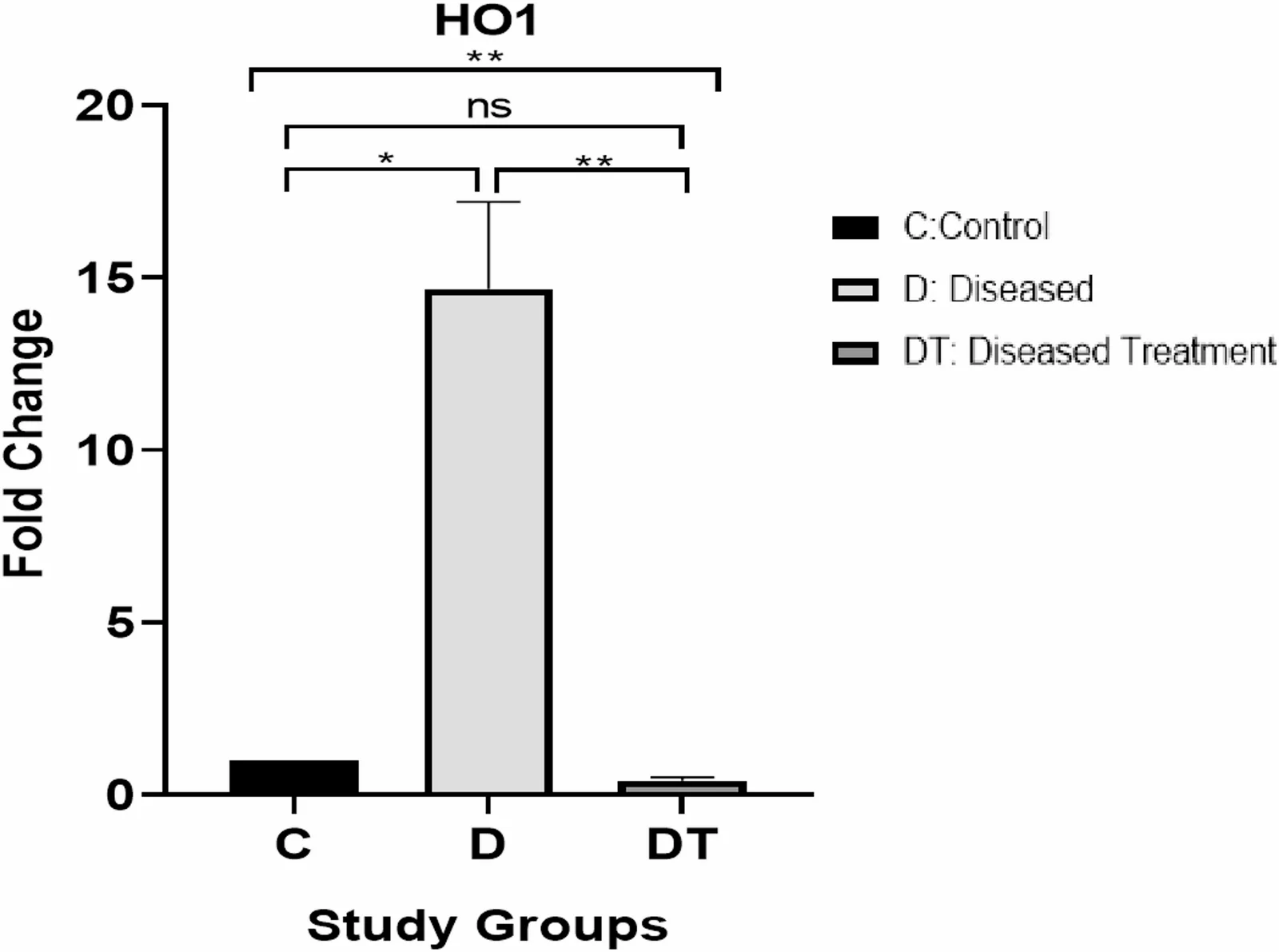
Changes in the mRNA expression of heme oxygenase 1 (HO-1). Graphical representation generated by Graphpad Prism, One Way ANOVA & multiple comparison test, HO-1 expression (ns = non-significant, *p < 0.05 ± SEM) in disease, (**p < 0.001 ± SEM) in treatment (*n* = 3; ns = nonsignificant)

The mRNA expression of HO-1 in the control group was normalized to 1, indicative of a basal level. The diseased group (VPA treatment) showed a significantly (*p* < 0.05) higher expression (16.98 ± 2.56) of HO-1, indicating severe hepatic oxidative stress. The diseased treated group (folates and VPA treatment) presented significantly (*p* < 0.01) lower levels (0.40 ± 0.03) of HO-1 than in the diseased group (Fig. 3).

**Fig. 4:**
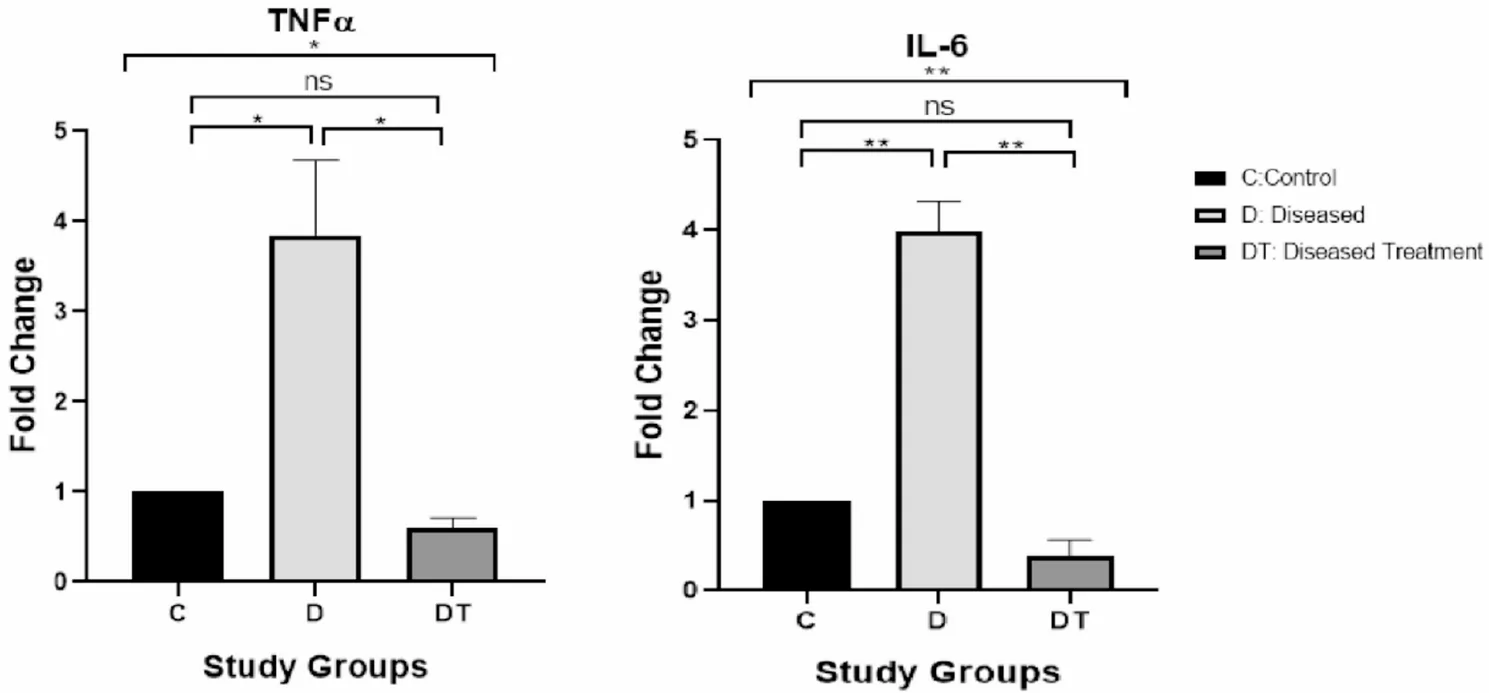
Changes in the mRNA expression of tumor necrosis alpha (TNF-α) and interleukin-6 (IL-6). Graphical representation generated by Graphpad Prism. One-way analysis of variance (ANOVA) & multiple comparison test was performed to analyze the significance. (*p < 0.05 ± SEM) in disease, (**p < 0.001 ± SEM) in treatment (*n* = 3; ns = nonsignificant)

The mRNA expression of TNF-α and IL-6 in the control group was normalized to 1. In the diseased group, there was a significant upregulation of TNF-α (7.36 ± 0.83, *p* ≤ 0.05) and IL-6(4.72 ± 0.34, *p* ≤ 0.01), compared to the control. However, in the treatment group (folates + VPA), TNF-α and IL-6 were found to be downregulated (0.97 ± 0.11, *p* ≤ 0.05) and (0.09 ± 0.2, *p* ≤ 0.01), respectively (Fig. 4).

### Bioactive folates exhibit strong binding affinity with KEAP1

The docking results indicated that both 5-MTHF and folinic acid have high binding affinities toward KEAP1. In the case of 5-mTHF, the best pose, with the highest score, was − 10.7 kcal/mol with 0.0 Å for RMSD (lower and upper bounds), indicating that this pose was highly stable and aligned well with the binding site, while the second and third poses had comparatively lower binding scores (-10.4 and − 10.3 kcal/mol) as shown in Table 3. Similarly, folinic acid shows strong binding with a top-scoring pose similar to that of 5-MTHF at -10.7 kcal/mol with a 0.0 Å RMSD, with poses 2 and 3 giving consecutive binding scores of -10.1 and − 9.8 kcal/mol. Overall, both ligands appear to have good stability and consistency in docking to Keap1.

**Table 3:** Binding affinities of Keap1 with 5-mTHF and folinic acid

Ligand	Binding Affinity kcal/mol	rmsd/ub	rmsd/lb
Keap1_5-mTHF	-10.7	0	0
Keap1_5-mTHF	-10.4	2.25	1.84
Keap1_5-mTHF	-10.3	1.61	1.43
Keap1_Folinic Acid	-10.7	0	0
Keap1_Folinic Acid	-10.1	2.37	1.47
Keap1_Folinic Acid	-9.8	2.17	1.84

The binding affinities of 5-mTHF plus folinic acid with the Kelch-like ECH-associated protein 1 (Keap1) were studied using molecular docking. Binding affinities are reported in kcal/mol, with more negative numbers indicating a stronger ligand protein contact. The RMSD/ub and RMSD/lb are the upper and lower bound of the root mean square deviation (RMSD) results, so they mainly indicate the degree of similarity of the docked conformations, even if the poses are slightly different

*5-mTHF* 5-methyltetrahydrofolate

**Fig. 5:**
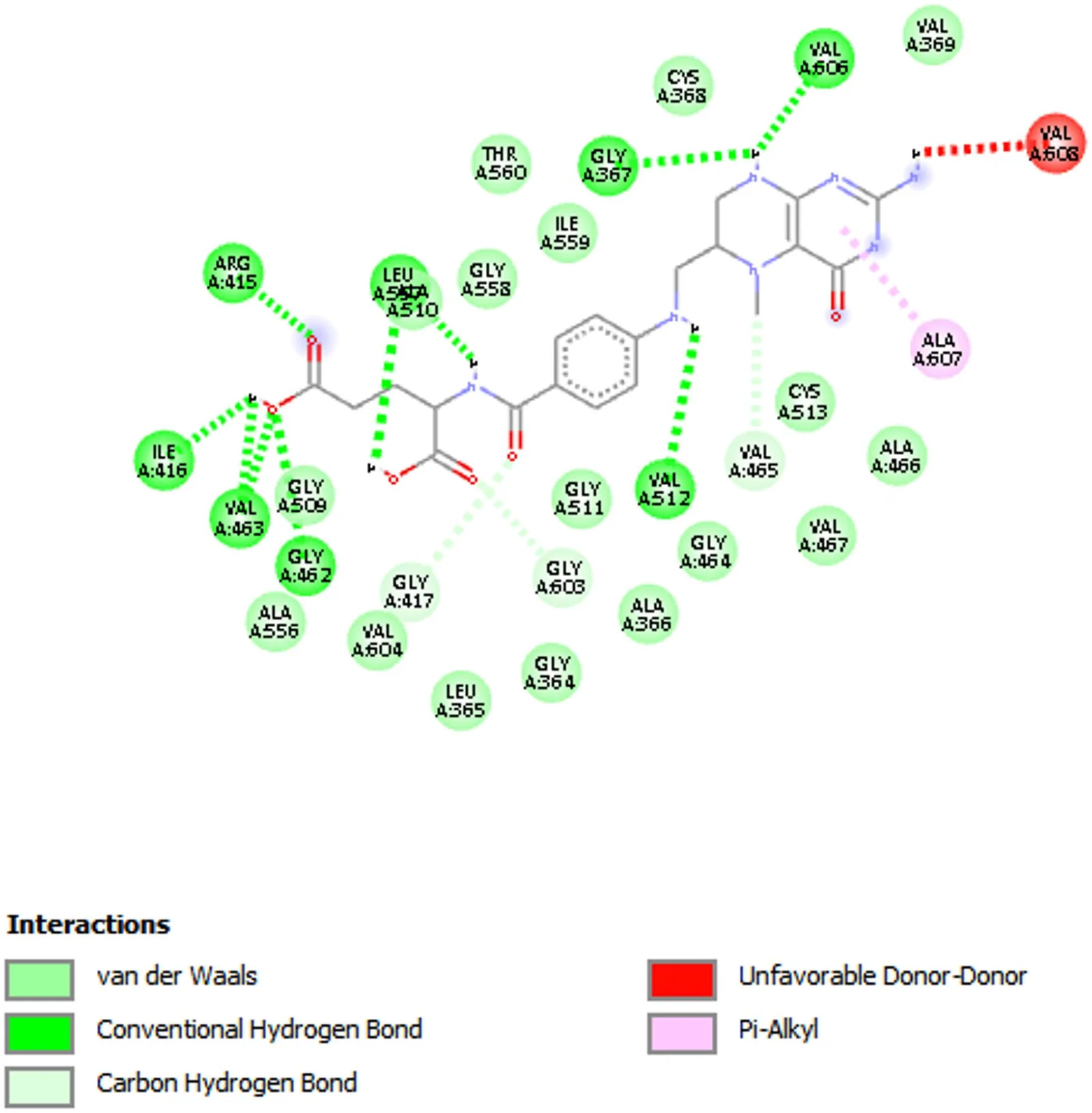
2-dimensional diagram of interaction between 5-methyl tetrahydrofolate and Kelch-like ECH-associated protein 1 (Keap1)

**Fig. 6:**
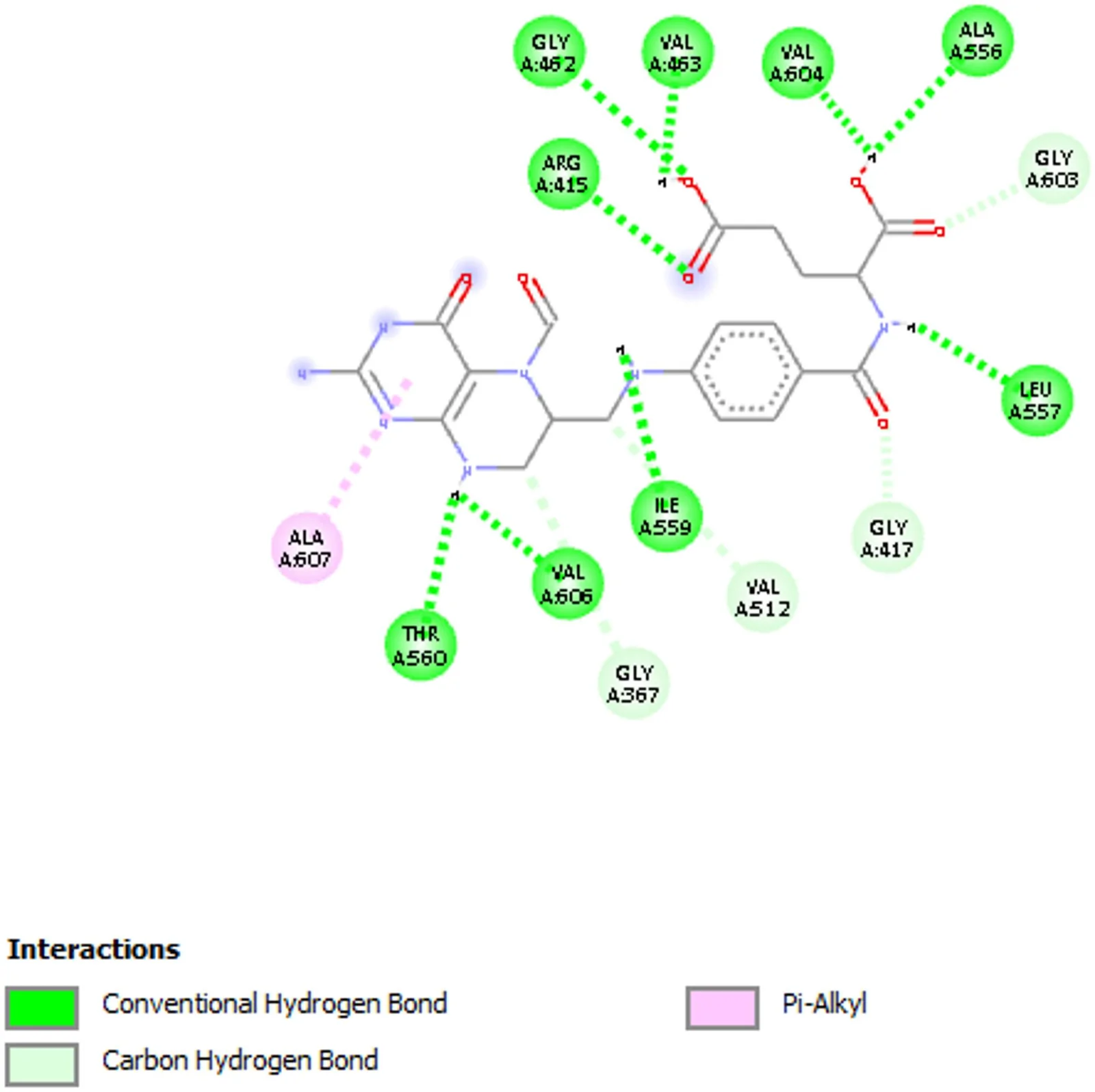
2-dimensional diagram of interaction between folinic acid and KEAP1

A detailed 2D interaction diagram (Fig. 5) showing binding of 5-MTHF with the Keap1 protein. The ligand interacts with many of the protein’s residues through hydrogen bonding, van der Waals interactions, and pi-alkyl interactions. There is one unfavorable donor-donor interaction with VAL A:506. These findings provide support from computer-aided modeling to substantiate the view that bioactive folates may act as modulators of oxidative stress by interacting directly with the KEAP1 protein. Correspondingly, Fig. 5 shows the detailed 2D interaction diagram of the binding of folinic acid with the Keap1 protein. The ligand binds with many protein residues via covalent hydrogen bonding, carbon-hydrogen bonding, and pi-alkyl interactions. Similarly, Fig. 6 shows the detailed 2D interaction diagram of the binding of folinic acid with the Keap1 protein. The ligand binds with many protein residues via covalent hydrogen bonding, carbon-hydrogen bonding, and pi-alkyl interactions.

## Discussion

The hepatoprotective and disease-modifying effects of folate supplementation in liver disorders, particularly NAFLD and hepatic steatosis, have been increasingly documented in experimental and mechanistic studies [3, 5]. However, previous studies have mainly employed synthetic folic acid [21], while the present study used bioactive folate derivatives folinic acid and 5-mTHF.

VPA is known to interfere with folate metabolism and impair the enzymatic conversion of synthetic folic acid into its active forms. Folinic acid (5-formyltetrahydrofolate) is a reduced folate that bypasses dihydrofolate reductase-dependent activation and directly replenishes intracellular tetrahydrofolate pools needed for nucleotide synthesis, DNA repair and cellular regeneration [22]. Folinic acid could be a promising candidate to protect hepatocytes from VPA induced oxidative stress, mitochondrial dysfunction and metabolic disturbances by maintaining reduced folates availability. In contrast, 5-mTHF is the biologically active, methylated form of folate and participates directly in one-carbon metabolism and homocysteine remethylation to methionine, thereby supporting S-adenosylmethionine (SAM) synthesis, DNA methylation, phosphatidylcholine production and hepatic lipid export [23]. Impaired phosphatidylcholine synthesis and defective very low-density lipoprotein (VLDL) secretion are major contributors to hepatic steatosis. Restoration of methylation pathways by 5-mTHF may attenuate hepatic lipid accumulation. The combined use of folinic acid and 5-mTHF may thus have synergistic hepatoprotective effects by targeting complementary parts of folate metabolism. Folinic acid replenishes intracellular reduced folate stores and supports the recovery of cells, while 5-mTHF directly restores. Hence, the combination of folinic acid and 5-mTHF may exert synergistic hepatoprotective effects by targeting complementary components of folate metabolism. In contrast, folinic acid restores the intracellular reduced folate pools and permits cell recovery, 5-mTHF on the other hand, directly restores methylation capacity and modulates hepatic lipid metabolism. These bioactive folates together may be more effective than synthetic folic acid alone in combating VPA-induced impairment of one-carbon metabolism, oxidative stress and hepatic lipid accumulation. Therefore, the use of bioactive folate derivatives in the present study is a novel hepatoprotective approach for VPA-induced NAFLD.

The present study showed that VPA exposure produced significant hepatic injury in rats as evidenced by marked increase in serum levels of ALT, ALP and total bilirubin indicating hepatocellular damage and impaired liver metabolic function. These findings are consistent with previous reports that VPA administration causes hepatotoxicity through mitochondrial dysfunction, impaired β-oxidation, and subsequent hepatic lipid accumulation [24].

Biochemical evidence of liver injury was confirmed by histopathological observations. The VPA group exhibited severe steatosis, fat droplet accumulation, distorted hepatic lobular architecture, and congested sinusoids, which are consistent with the NAFLD pathology. Folate-treated rats showed almost normal hepatic architecture, absent steatosis, and sinusoidal spacing improved with evidence of hepatoprotection. In addition, NAS showed complete resolution of fatty liver changes in the folate-treated group (NAS = 0) vs. mild NAFLD in the VPA group (NAS = 2). These findings in rats corroborate several previous studies stating the role of folates in regulating hepatic lipid metabolism, SAM/SAH balance, and preventing fat accumulation [2, 17].

**Fig. 7:**
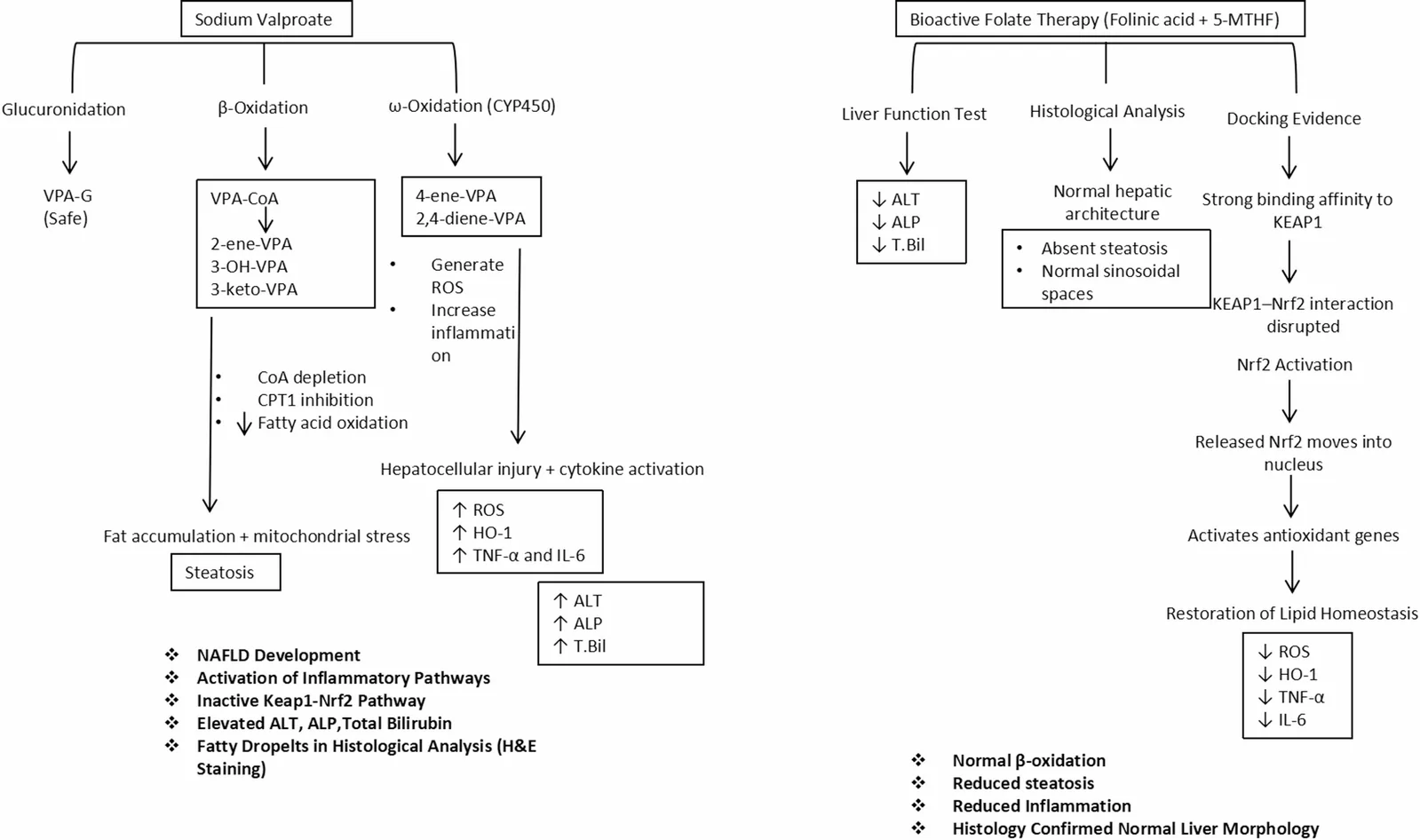
Mechanistic diagram illustrating how valproic acid disrupts hepatic lipid metabolism and induces oxidative-inflammatory injury, and counterreaction of bioactive folate therapy. Graphical Abstract: VPA accelerates the development of NAFLD, which is induced by increased oxidative stress, inflammatory response and hepatic steatosis. Folate supplementation increases KEAP1 binding with high docking energy of ~ − 10.7 kcal/mol, leading to the activation of NRF2 signaling. The activation of NRF2 upregulates the expression of antioxidant and cytoprotective genes in hepatocytes, thereby reducing oxidative stress and inflammation and improving hepatic function

At the molecular level, the study showed that VPA induced hepatic oxidative damage and activated inflammatory response as indicated by high mRNA expression of HO-1 and the inflammatory cytokines (TNF-alpha and IL-6). Moreover, the study indicated that bioactive folates downregulated hepatic oxidative stress and inflammation markers. VPA exposure significantly upregulated HO-1 and inflammatory cytokines, signifying oxidative injury and liver inflammation. Folate-treated rats showed a significant reduction in HO-1 and inflammatory cytokines, highlighting the anti-inflammatory and antioxidative potential of bioactive folates. These findings align with reports that state that methyl donors from folate restore one-carbon metabolism and decrease hepatic inflammation by enhancing NF-κB and Nrf2 signaling [14,15]. Figure 7 represents the graphical representation of the proposed pathway for the current study.

Supporting the protective role of bioactive folates, our recent study demonstrated that supplementation with 5-mTHF and folinic acid ameliorated VPA-induced placental injury by preserving placental architecture, restoring junctional zone cell density, and significantly reducing inflammatory mediators (TNF-α and IL-6) as well as oxidative stress marker expression (HO-1)[20]. These results support the strong cytoprotective, anti-inflammatory and antioxidative effects of bioactive folates against VPA-induced tissue injury. The protective role may be attributed to the complementary metabolic functions of the selected folate derivatives in hepatic one-carbon metabolism, methylation reactions, antioxidant defense, and maintenance of lipid balance. Folates have this central place in nucleotide synthesis, supplying methyl donors, and managing homocysteine metabolism. These parts are tightly tied to hepatic metabolic integrity, and also to the prevention of steatosis [25]. When the liver is affected by VPA, it tends to show higher oxidative stress, mitochondrial damage, and disrupted lipid metabolism, which facilitates the development and progression of NAFLD. In the current study, bioactive folates, might provide therapeutic benefits, because they go around some enzymatic activation stages that could be less efficient during hepatic dysfunction and oxidative stress [26].

Moreover, having enough folate availability has been connected with better methylation capacity, plus regulation of genes tied to lipid metabolism and inflammatory signaling routes [27]. Therefore, the rationale for co-administering folate derivatives in the present work was to offer a broader metabolic coverage across multiple, inter-related pathways rather than focusing on any one single pathway or target. However, we are not able to define the precise contribution of each folate derivative in this experimental setting. Future work should employ separate treatment groups for each bioactive folate compound, so we can tease apart their individual roles, and also see whether any independent, or even synergistic effects show up in VPA induced hepatic steatosis.

Molecular docking studies in this rat-based experimental framework showed strong binding affinity of folinic acid and 5-mTHF towards KEAP1 suggesting possible activation of the Nrf2-KEAP1 antioxidant pathway. The binding scores of 10.7 kcal/mol for both the ligands indicate the stabilization of Nrf2 signaling and reduction of oxidative damage as observed in the VPA treated rats. Previous in silico studies have shown that folate derivatives bind to KEAP1 and sustain Nrf2-mediated protection [13, 28]. Taken together, in silico outcomes alongside the in vivo histopathological and molecular evidence, they fit together and add mechanistic support for the potential hepatoprotective effects attributed to these bioactive folates. Still, even if the docking data strengthen the biological plausibility of the proposed signaling route, they remain predictive only and need in vivo experimental confirmation. So overall, even though the study helps explain the mechanism within this rat model, any direct correlation to other species or to clinical use should be taken carefully, because there are species-specific differences, plus computational modeling also has its usual limitations.

Interestingly, some experimental findings indicated that oxidative stress could decrease the uptake of folic acid by downregulating folate transporters and receptors [29]. Moreover, disturbances in folate homeostasis can dysregulate autophagy, leading to hepatic lipid accumulation and therefore it appears, under conditions of metabolic stress, that folate metabolism is incapable of supporting antioxidant and lipid-regulating pathways adequately [18]. On the contrary, bioactive folates circumvent this enzymatic conversion and are therefore directly accessible for cellular uptake, as demonstrated in this study.

Fatty acid (FA) metabolism is tightly controlled by multiple cellular pathways, which are important for maintaining hepatic lipid homeostasis and energy balance. In that whole picture, peroxisomes seem to be playing an important role in the oxidation of very-long-chain fatty acids, so they can prevent excessive lipid accumulation within hepatocytes. Disruption of fatty acid oxidation pathways has been associated with increased oxidative stress30 and hepatic steatosis [30]. Some previous work has also reported that VPA can interfere with fatty acid metabolism and mitochondria performance, and that this may facilitate lipid accumulation and liver damage [31]. Moreover, transcriptional regulators like peroxisome proliferator-activated receptor alpha (PPAR-α) have been associated with the regulation of genes involved in fatty acid oxidation [32], but those pathways were not evaluated in this study.

Taking into account the hepatic lipid accumulation observed in the present study and the histopathologic modifications, one can hypothesize that dysregulation of fatty acid oxidation is involved in the metabolic alterations induced by VPA [33]. However, the present study did not measure markers for PPAR signaling, peroxisomal function, or beta-oxidation, so the role of these specific mechanisms is speculative. So, future work will require molecular analyses of lipid metabolic pathways. This will help to establish whether alterations in PPAR-α activity or peroxisomal fatty acid metabolism, are indeed involved in the hepatic effects observed in this model.

### Limitations

The current study gave biologically relevant and mechanistic insight into drug-induced hepatic steatosis. It also helped show that bioactive folates may have hepatoprotective effects. However, there are some limitations. The sample size was relatively small, and only one animal species and only one gender were used, so the broader generalizability might be limited. The number of animals was picked using previous exploratory in vivo work, keeping in mind the ARRIVE guidelines, together with the 3Rs, so animal use was minimized. Still, having a bigger cohort and including a folate-only control group would have strengthened statistical power and made the mechanistic reading clearer. But this work was meant more as a preliminary investigation, mainly to look at the hepatoprotective potential of bioactive folates against VPA-induced NAFLD. Also, folinic acid and 5-methyltetrahydrofolate doses that were used here were already set in our previous work as safe and human-equivalent. From an ethical angle tied to reducing animal numbers, we did not add a folate-only group.

### Future direction

Future studies should continue to explore the protective mechanisms including Nrf2-mediated antioxidant signaling, folate receptor-dependent transport, changes in SAM/SAH ratio, and a more complete lipid metabolomic profiling. Moreover, the role of gut-liver axis and fecal microbiota is still a major open question in the present research. Gut microbiota are known to regulate folate availability, one-carbon metabolism, nutrient processing and also host methylation capacity. Dysbiosis is strongly associated with the progression of NAFLD via pathways such as increased intestinal permeability, endotoxemia, bile acid dysregulation and hepatic inflammation. VPA-induced systemic metabolic changes may indirectly influence gut microbial composition and gut-liver communication pathways, although intraperitoneal administration of VPA circumvents direct exposure to the gastrointestinal tract. In the same vein, microbiota-dependent changes in folate metabolism may have implications for the biological utilization of bioactive folates. These interactions were not measured directly and represent a mechanistic limitation. Therefore, more studies using fecal microbiota profiling and metabolomic analyses might help to better understand the gut-liver-folate axis in VPA-induced NAFLD or to clarify the hepatoprotective mechanisms of bioactive folates.

### Clinical implications

Though this is an exploratory study, it still sets a clinical understanding highlighting the importance of the use of biologically active forms of folates during VPA treatment. The main take-home message from this study could matter clinically for patients under an extended duration of VPA treatment, especially when NAFLD is present or developing. A strategy that uses bioactive folate supplementation, and particularly the combination of folinic acid with 5-mTHF, might proffer a safer and more effective prevention of impaired hepatic function, steatosis, and related inflammatory and oxidative injury compared with conventional folic acid. By skipping or bypassing metabolic bottlenecks tied to MTHFR polymorphisms or compromised hepatic conversion, bioactive folates may improve outcomes in those patients who are more vulnerable. Their affinity for the KEAP1 protein also suggests a possibility of an enhanced endogenous defense system against oxidants. Taken together, this understanding supports the rationale for clinical trials that test bioactive folates as an add-on for valproate-treated epileptic and psychiatric patients.

## Conclusions

In conclusion, the present study provides the first in vivo evidence that combined supplementation of two bioactive folates, folinic acid and 5mTHF, can ameliorate VPA-induced NAFLD by modulating oxidative stress, inflammation, and disturbances in folate metabolism. Our findings point to a new concept around targeting one-carbon metabolism along with antioxidant signaling pathways, which could become a practical direction for preventing drug-induced liver damage. Even though this is preliminary and will need further validation, it still establishes a foundation for translational research on hepatoprotection in people receiving long-term VPA.

## Data Availability

The data is available from the corresponding author upon request.

## Abbreviations

5-mTHF: 5-methyltetrahydrofolate
ALP: Alkaline phosphatase
ALT: Alanine aminotransferase
cDNA: Complementary DNA
CBZ: Carbamazepine
DGAT2: Diacylglycerol O-acyltransferase 2
DT: Diseased treated
EDTA: Ethylenediaminetetraacetic acid
FRα: Folate receptor alpha
H&E: Hematoxylin and eosin
HO-1: Heme oxygenase-1
IFCC: International Federation of Clinical Chemistry
IL-6: Interleukin-6
KEAP1: Kelch-like ECH-associated protein 1
LFT: Liver function test
LTG: Lamotrigine
MTHFR: Methylenetetrahydrofolate reductase
NAFLD: Non-alcoholic fatty liver disease
NAS: NAFLD Activity Score
NF-κB: Nuclear factor kappa-light-chain-enhancer of activated B cells
Nrf2: Nuclear factor erythroid 2-related factor 2
NUST: National University of Sciences & Technology
PFA: Paraformaldehyde
RMSD: Root-mean-square deviation
RNA: Ribonucleic acid
RT-PCR: Reverse transcription polymerase chain reaction
SAM: S-adenosylmethionine
SAH: S-adenosylhomocysteine
SD: Standard Deviation
SEM: Standard error of the mean
TNF-α: Tumor necrosis factor-alpha
THF: Tetrahydrofolate
VLDL: Very low-density lipoprotein
VPA: Valproic acid
